# Intracranial infections of *Candida albicans* and *Mycobacterium intracellulare* associated with CARD9 deficiency: case report and review of literature

**DOI:** 10.3389/fimmu.2025.1648436

**Published:** 2025-09-17

**Authors:** Congchen Tang, Chao Chen, Xiaoju Lv, Yi Xie, Li Xiong, Jiangchao Long, Hui Ye

**Affiliations:** ^1^ Center of Infectious Diseases, West China Hospital, Sichuan University, Chengdu, Sichuan, China; ^2^ Department of Infectious Diseases, The Second People’s Hospital of Yibin, Yibin, Sichuan, China; ^3^ Laboratory Medicine Department, West China Hospital, Sichuan University, Chengdu, Sichuan, China; ^4^ Intensive Care Unit, Care Alliance Hetai Rehabilitation Hospital of Chengdu Tianfu New Area, Chengdu, Sichuan, China

**Keywords:** CARD9, Candida albicans, Mycobacterium intracellulare, immune deficiency, case report

## Abstract

**Background:**

Invasive candidiasis, most commonly caused by *Candida albicans*, poses a significant mortality risk and is challenging to treat. Non-tuberculous mycobacterial infections are opportunistic and linked to immune impairment. Caspase recruitment domain-containing protein 9 (CARD9) represents a class of proteins that incorporates the caspase recruitment domain, and its deficiency follows a strict autosomal recessive inheritance pattern, resulting in an impaired immune response.

**Case presentation:**

A 51-year-old male who was admitted to the hospital 3 years ago because of recurrent fever accompanied by headache. The causative factor remains elusive and symptomatic treatment yielded unsatisfactory results. Next-generation sequencing (NGS) of cerebrospinal fluid (CSF) identified the fungus as *C. albicans*. Following antifungal therapy, the patient experienced relief from fever and headache; however, he subsequently developed a hydrocephalus. CSF culture indicated NTM—*Mycobacterium intracellulare*, prompting the initiation of anti-NTM treatment. Given the recurrent infections, we collected peripheral blood for whole exome sequencing, which revealed a CARD9-deficient homozygote with a new mutation site identified as c.175C>T (p. Arg59Trp). The patient was hospitalized on 8 occasions for diagnostic assessment and treatment. Presently, antifungal treatment has been discontinued after 9 months of therapy, while anti-NTM therapy is being maintained, with the patient reporting no fever or other discomforts.

**Conclusion:**

The c.175C>T (p. Arg59Trp) mutation is a novel CARD9 gene mutation and is probably damaging. Clinicians should consider immune impairment as a contributing factor in the management of fungal infections among non-HIV/AIDS patients. For such patients, conducting multiple CSF and blood cultures and employing new technologies such as NGS are advisable. Treatment of NTM and *C. albicans* requires personalized treatment plans. Moreover, the long-term follow-up should not be overlooked.

## Introduction

The incidence of systemic fungal infections has notably surged in recent years, attributable to factors such as the increasing use of intravenous cannulas and implantable medical devices, alongside an increasing immunosuppressed population stemming from various causes. According to a study (1), fungal infections affect approximately 1 billion individuals, resulting in over 1.5 million deaths globally. Opportunistic fungal infections have emerged as an important cause of mortality among hospitalized patients, presenting a substantial public health challenge with significant clinical and socioeconomic ramifications that require urgent attention (2). Among the pathogens responsible for opportunistic infections, *Candida* stands out as particularly noteworthy, given its status as the most prevalent bloodstream infection in tertiary care hospitals (2). Furthermore, invasive candidiasis poses significant treatment challenges and has a high mortality rate, with *C. albicans* being the most frequently encountered pathogen.

### Candida albicans

*C. albicans* can exist harmlessly in symbiosis with the host or transition to an opportunistic pathogen. It naturally colonizes various parts of the human body, including the oral cavity, intestinal tract, vagina, and skin. The growth of *C. albicans* is typically controlled by the host immune system and regulatory mechanisms facilitated by normal microbiota (3). However, when this delicate balance is disrupted, *C. albicans* proliferates excessively and becomes more virulent. Factors influencing clinical manifestations include the specific site affected, route of infection, intrinsic characteristics of the pathogen, and underlying health conditions of the patients. As a result, the clinical presentations can vary widely, ranging from mild superficial infections to severe systemic illnesses. Therefore, accurate diagnosis and appropriate treatment are crucial for managing *C. albicans* infection. A 12-year retrospective analysis of autopsies conducted at the University of Kentucky Medical Center revealed that 54% of patients with positive *C. albicans* cultures had invasive candidiasis. Among these cases, the proportion of infected sites included the kidneys (80%), the brain (52%), and the heart (48%) (4). *C. albicans* infection of the central nervous system(CNS), previously thought to be rare, appears to be increasing in incidence; in most cases, the disease typically originates from fungal hematogenous dissemination, where the fungus spreads through the bloodstream from distant primary sites of infection (4) and occurs predominantly in individuals with certain types of immunodeficiencies, often leading to neurological sequelae or death if not diagnosed and treated in a timely manner (5). The main receptor families involved in *C. albicans* recognition are C-type lectin receptors (CLR), RIG I-like receptors (RLR), NOD-like receptors (NLR), and Toll-like receptors (TLR), which recognize different pathogen-associated molecular patterns (PAMPs) (6). The successful clearance of *C. albicans* from host tissues largely depends on the phagocytosis of this fungal pathogen by innate immune cells (i.e., macrophages, neutrophils, and dendritic cells). Exposure to β-glucan of *C. albicans* triggers proinflammatory innate immune responses among CLRs, particularly those dependent on DECTIN1/CLEC7A (C-type lectin domain family 7 member A) (7, 8). When this pathway is impaired, it reduces the body’s clearance rate of Candida albicans.

### Non-tuberculous Mycobacteria

Non-tuberculous Mycobacteria (NTM), which are members of the *Mycobacterium* genus, excluding *Mycobacterium tuberculosis* and *Mycobacterium leprae*, are ubiquitous organisms commonly found in natural environments such as water and soil (9). Although NTM encompasses more than 190 species, only a small subset is associated with human diseases, with lung diseases being the most prevalent (10). CNS infections are relatively rare, and NTM is traditionally viewed as an opportunistic infection closely linked to immune impairment. Disruption of the IFN-γ/IL-12 signaling pathway has been identified as the primary cause of disseminated NTM infection (11). A study on the US national managed care claims database showed that the incidence rate of NTM disease in the population ranges from 3.1 to 4.7 per 100,000 person-years (12), with *Mycobacterium avium complex* (MAC: *M. avium*, *Mycobacterium intracellulare* and *Mycobacterium chimaera*) being the most common NTM species causing CNS diseases (13).

### CARD9

Caspase recruitment domain-containing protein 9 (CARD9) is a class of proteins containing the caspase recruitment domain (14), and the gene encoding CARD9 is located on chromosome 9 at position q34.3 with 13 exons. Human CARD9 transduces signals downstream of different CLRs (e.g., DECTIN1/CLEC7A, DECTIN2/CLEC6A, DECTIN3/CLEC4D, and MINCLE/CLEC4E) from fungal constituents through Syk activation of ITAM, resulting in activation of the NF-κB and MAPK pathways as well as proinflammatory cytokine production, thereby triggering a comprehensive antifungal immune response (15). CARD9 deficiency follows an autosomal recessive pattern and results in an inadequate immune response to fungi, leading to subsequent infection. However, the heterozygotes did not exhibit abnormal phenotypes. CARD9 deficiency has been reported worldwide, including but not limited to China (16), Iran (17), Canada (18), Algeria (19), Morocco (20), Tunisia (21), and the United States (22). The three regions with the most reports are China, Algeria, and Iran. Among them, *Trichophyton* and *Candida* are the two most prominent pathogenic fungi (23).

## Case report

**Figure 1:**
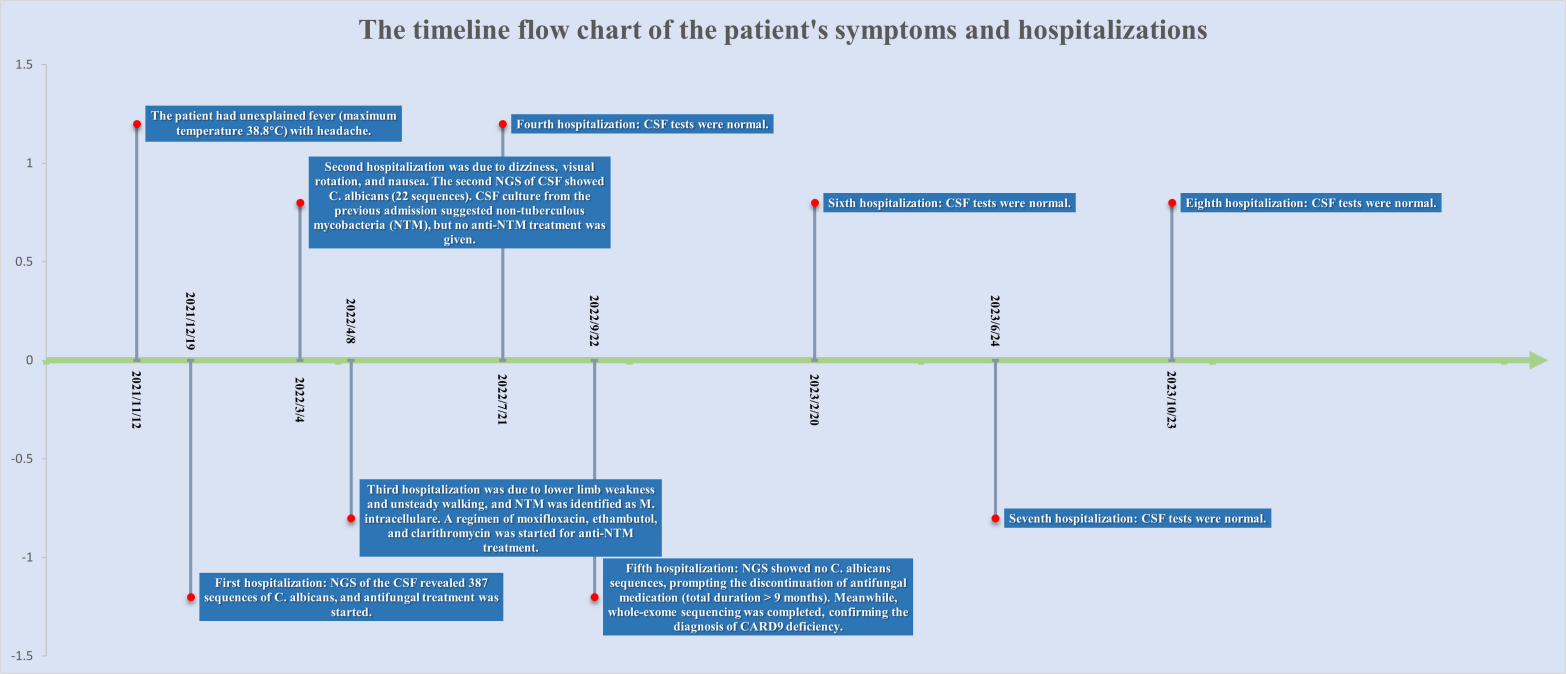
The timeline flow chart of the patient’s symptoms and hospitalizations.

The patient is a 51-year-old male residing in Sichuan Province, China. His parents were Han Chinese first cousins, and his mother died of lung cancer in 2003.He had healthy siblings and a son with no clear history of genetic or familial diseases. In 2018, he was diagnosed with lumbar spine tuberculosis, underwent surgical excision, and received antituberculosis treatment for more than 1 year, achieving a clinical cure. In November 2021, he experienced an unexplained fever (highest temperature 38.8 °C) accompanied by headache. He visited the local hospital twice, and various tests showed no significant abnormalities. Empirical treatment was administered, however, the fever persisted. The timeline flow chart of the patient’s symptoms and hospitalizations is shown in **Figure 1**.

### The first hospitalization to the Infectious Diseases Center of West China Hospital

**Figure 2:**
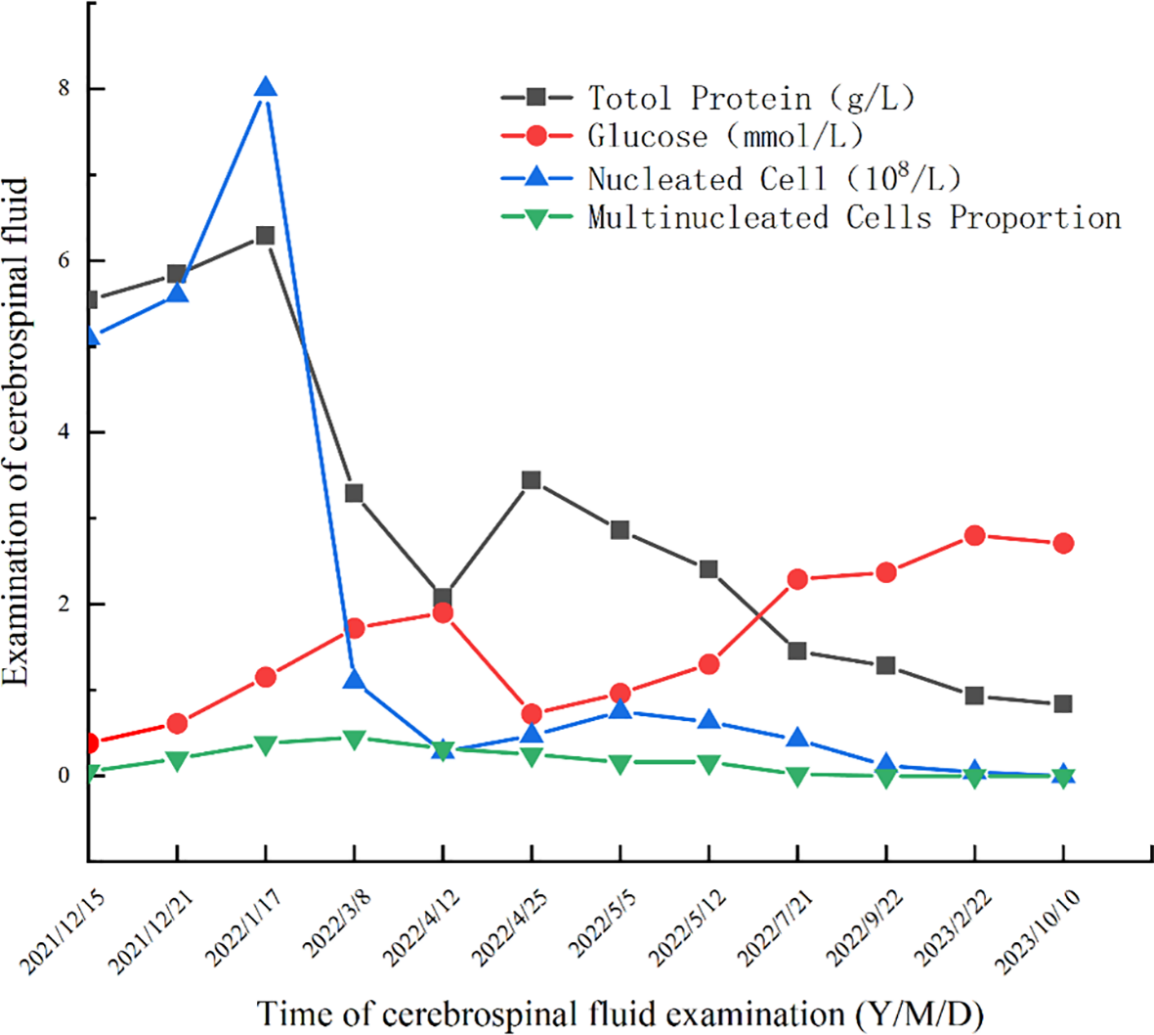
Cerebrospinal fluid examination results.

On December 14, 2021, upon arrival at our hospital, the patient underwent a physical examination that revealed clear consciousness, chronic facial features, absence of neck stiffness, Kernig’s sign, and Brudzinski’s sign. No palpable lymph node enlargement was observed, and cardiovascular, pulmonary, or abdominal examination revealed no obvious abnormalities. Further investigations included CD4/CD8 cells, neutrophils, HIV, TB-IGRA, TB-DNA, CMV-DNA, EBV-DNA, blood CrAg, tumor markers, PCT, and erythrocyte sedimentation rate, all of which showed no significant abnormalities. In the initial blood test, the only notable increase observed was in IgE level, which was 443 IU/mL. Enhanced MRI of the head revealed a bilateral frontal lobe and small ischemic lesions in the left central semiovale. The cerebrospinal fluid (CSF) analysis was negative for CrAg, ink staining, smears, and culture. However, protein and nucleated cell counts in the CSF were significantly elevated, whereas glucose levels were decreased (**Figure 2**). Despite empirical antimicrobial treatment, the patient’s temperature remained elevated. On December 21, 2021, the CSF was rechecked, showing similar results, with increased protein and nucleated cells and decreased glucose levels (**Figure 2**). Autoimmune encephalitis antibodies were negative, meanwhile also ruled out the possibility of connective tissue disease; however, Next-generation sequencing (NGS) of the CSF revealed 387 sequences of *C. albicans*. Considering the abnormal CSF test results and fever, *C. albicans* meningitis was diagnosed. Consequently, fluconazole (400mg qd) and flucytosine (1.5g qid) were administered on December 23, 2021. After approximately 10 days of treatment, the CSF did not show significant improvement (**Figure 2**). Therefore, amphotericin B (40mg qd) was added to the treatment regimen. However, owing to acute renal function injury, amphotericin B was discontinued and replaced with voriconazole (200mg bid) and flucytosine (1.5g qid) before discharge on January 24, 2022.

### The second hospitalization to the Infectious Diseases Center of West China Hospital

After discharge, the patient adhered to antifungal therapy and the headache and fever resolved. However, he was readmitted on March 4, 2022, due to dizziness, visual rotation, and nausea. CSF re-examination showed decreased protein and nucleated cell counts and increased glucose levels compared to the previous admission. CSF culture from the previous admission suggested non-tuberculous mycobacteria (NTM); however, the identification and drug sensitivity results were inconclusive. The second NGS of CSF showed C*. albicans* (22 sequences), *Aspergillus* (6 sequences), and *Torque teno virus* (4 sequences). Magnetic resonance imaging (MRI) of the head revealed meningeal thickening and ventricular enlargement with hydrocephalus. As CSF results improved, anti-NTM treatment was not administered. Antifungal therapy was continued and the patient was discharged.

### The third hospitalization to the Infectious Diseases Center of West China Hospital

**Figure 3:**
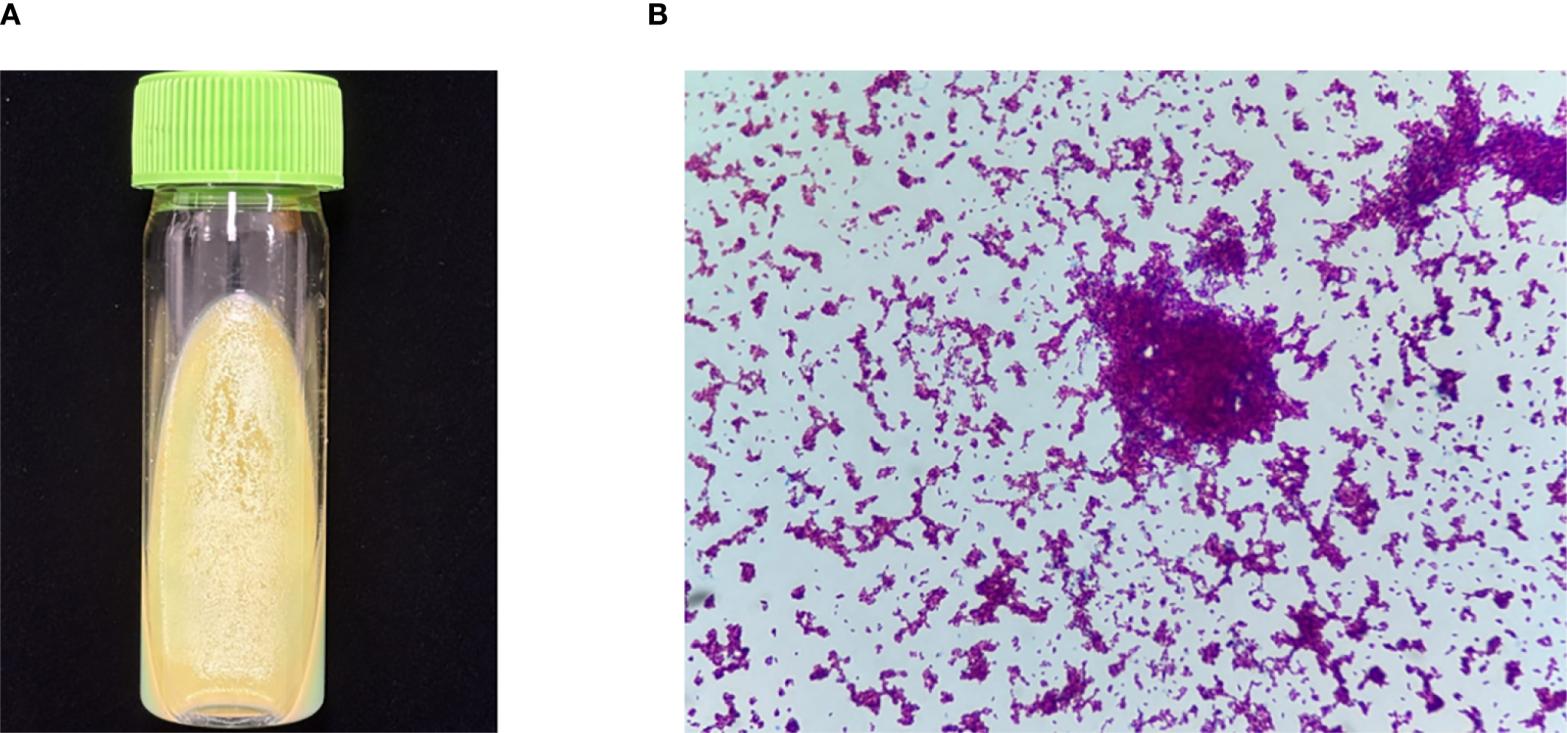
Culture and smear of M. intracellulare. **(A)** Culture of M. intracellulare (Lowenstein Jensen media, 35°C, 6 weeks). **(B)** Smear of M. intracellulare (Ziehl-Neelsen staining, original magnification×1000).

The patient was readmitted to the hospital on April 8, 2022, for lower limb weakness with unsteady walking. Enhanced MRI of the head showed enlarged ventricular hydrocephalus with interstitial edema, which was aggravated from the last follow-up. CSF was reviewed, the amount of protein and nucleated cells was significantly decreased, and glucose was increased (**Figure 2**). The symptomatic treatment of hydrocephalus yielded limited efficacy. On April 11, with the assistance of neurosurgery, lumbar large-pool drainage was optimized, resulting in a daily drainage flow of approximately 120mL. Subsequently, on April 18, the patient developed high fever (39 °C) accompanied by loss of consciousness. Blood and CSF cultures revealed the presence of *Klebsiella oxytoca*, leading to the initiation of effective antibacterial therapy. Finally, NTM was identified as *M. intracellulare* (**Figure 3**). In accordance with the drug sensitivity results, the patient was administered a regimen of moxifloxacin (400mg qd), ethambutol (750mg qd), and clarithromycin (500mg bid) for anti-NTM treatment.

### Follow-up hospitalization

Subsequent hospitalizations were aimed at assessing and monitoring the treatment efficacy. CSF analysis was conducted during the 4th hospitalization in July 2022, 5th hospitalization in September 2022, 6th hospitalization in February 2023, 7th hospitalization in June 2023, and 8th hospitalization in October 2023. The results showed normalization and stabilization (**Figure 2**). Additionally, NGS of the CSF during the 5th hospitalization revealed no *C. albicans* sequences, prompting the discontinuation of antifungal medication after September 2022 (total duration>9 months). Both the patient and his son underwent exome sequencing, which indicated that the patient was homozygous for CARD9 deficiency, with a mutation site at c.175C>T (p. Arg59Trp). The gene mutation was predicted to be “probably damaging” with a score of 1.000 by PolyPhen-2 (http://genetics.bwh.harvard.edu/pph2/).

Genomic DNA from the proband, father, two siblings, and son was amplified by polymerase chain reaction (PCR) using the following primers for Sanger sequencing of the identified CARD9 missense mutation (p. Arg59Trp): forward, 5’-F: GCCCTCAGCTCCTCTGCCCATTCCA-3’; reverse, 5’-GGACCCAACACCACTGCCCGCTCC-3’, with an annealing temperature of 60 °C. All gene mutations are shown in **Figure 4①**. His father and son are heterozygotes; he and his brother are homozygotes; and his sister is wild type.

**Figure 4:**
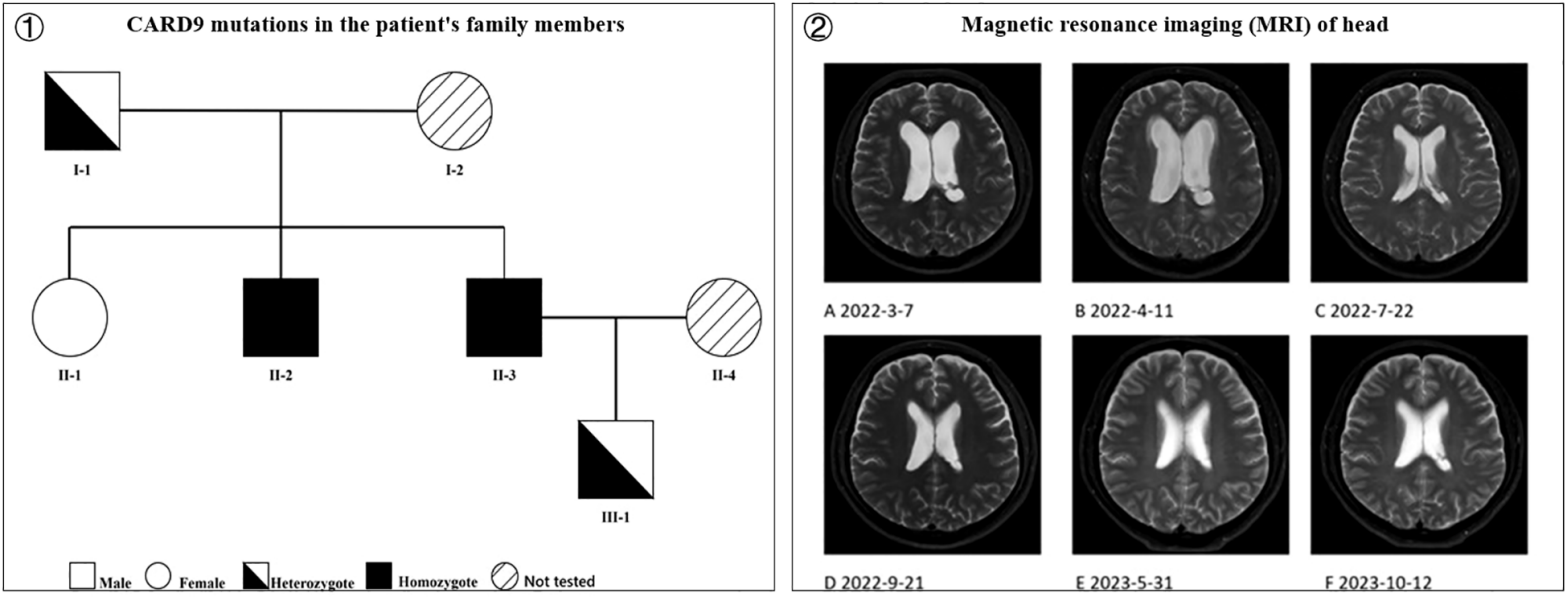
**①** CARD9 mutations in the patient’s family members (I-1 is the patient’s father, heterozygote; I-2 is the patient’s mother, not tested; II-1 is the patient’s sister, wild type; II-2 is the patient’s brother, homozygote; II-3 is the patient, homozygote; II-4 is the patient’s wife, not tested; III-1 is the patient’s son, heterozygote) and **②** Magnetic resonance imaging (MRI) of head. **(A)** Thickened meninges, enlarged ventricles with fluid; **(B)** Enlarged ventricles with fluid and interstitial hydrocephalus, worse than before; **(C)** Significant reduction in hydrocephalus and interstitial hydrocephalus compared to before; **(D)** No significant change in ventricles compared to before; **(E)** Enlarged triventricles and bilateral lateral ventricles, without significant fluid; **(F)** No significant change compared to previous scans).

To date, the patient has discontinued anti-NTM medications for nearly a year (discontinued in August 2024, duration 2 years) and antifungal drugs (discontinued in September 2022, duration over 9 months) for more than two years, with no fever, headache, dizziness, or physical impairment. Head MRI examinations conducted during this period indicated gradual disappearance of the hydrocephalus (**Figure 4②**).

## Review of literature

### Genetic vulnerability to fungal infections

Fungal infections are prevalent in individuals with immune dysfunction. However, in clinical practice, greater emphasis is typically placed on fungal infections in HIV/AIDS patients, potentially overshadowing the significance of immune dysfunction-related fungal infections in non-HIV/AIDS patients. In addition to CARD9, this review discusses several other genetic factors associated with the susceptibility to fungal infections, including high IgE syndrome (HIES), Autoimmune Polyendocrinopathy–Candidiasis–Ectodermal Dystrophy (APECED) syndrome, Dendritic cell-associated C-type lectin-1 (Dectin-1) deficiency, STAT1 mutations, and IL-17 mutations. These factors collectively constitute the primary immunodeficiency disorders (PIDD).

### High IgE syndrome

The clinical features of HIES include markedly elevated serum IgE levels, eczema, and recurrent skin and lung infections. HIES presents two distinct genetic patterns, autosomal dominant inheritance (AD) and autosomal recessive inheritance (AR). Among these, AD-HIES is the most prevalent genetic form. It exhibits unique clinical manifestations, including distinctive facial features, persistent deciduous teeth, and skeletal/connective tissue abnormalities, which are infrequently observed in AR-HIES patients (24). Although the precise immunodeficiency mechanisms and underlying causes of HIES remain unclear, it is evident that immune system dysregulation contributes significantly to the pathogenesis of the disease. This dysregulation primarily stems from the Th1/Th2 imbalance and aberrant production of cytokines and chemokines (25).

### APECED syndrome

Autoimmune Polyendocrinopathy–Candidiasis–Ectodermal Dystrophy (APECED) Syndrome, also referred to as Autoimmune Polyendocrinopathy Syndrome I (APS I), is a rare autosomal recessive autoimmune disorder arising from a single gene mutation. It is distinguished by autoimmune polyendocrinopathy, candidal infection susceptibility, and ectodermal dystrophy (26).Caused by loss-of-function variants of the autoimmune regulatory gene AIRE, occurring in either single or double allele forms, which compromises the thymus’s ability to perform negative selection against autoreactive T cells (27)and disrupts peripheral B cell tolerance (28). Research has demonstrated that JAK inhibitors are effective in managing these condition (29).

### Dectin-1 deficiency

Dendritic cell-associated C-type lectin-1 (Dectin-1), also recognized as the β-glucan receptor, represents an emerging PRR within CLR family. It identifies ligands independently of calcium ions (Ca^2+^), and primarily facilitates the connection between innate and adaptive immunity. Dectin-1 is predominantly expressed in dendritic cells, macrophages, and neutrophils and recognizes ligands from the PAMP family, such as β-1,3-glucans. Human Dectin-1 deficiency is associated with vaginal candida infections (30). Additionally, Dectin-1-deficient mice are susceptible to Candida infections (31).

### STAT1 mutation

Signal Transducer and Activator of Transcription (STAT) proteins serve as crucial components of both innate and adaptive immune responses against pathogenic microorganisms. Upon cytokine binding to hematopoietic receptors, signaling is initiated through Janus kinases (JAK1, JAK2, JAK3, and Tyk2) and subsequently via STAT proteins (STAT1, STAT2, STAT3, STAT4, STAT5A, STAT5B, and STAT6) (32). The molecular pathway mediated by JAK-STAT1 has garnered significant attention in recent years because of its involvement in the IFN-γ-mediated host defense against intracellular pathogens. A mutation in the coiled-coil domain (CC-D) of STAT1 was identified in an index patient from Ukraine, and has been observed in numerous patients from different familial backgrounds worldwide (33). Individuals with functional acquired mutations affecting STAT1 CC-D are predisposed to develop mucosal, skin, and nail candidiasis (34).

### IL-17 mutation

IL-17 is a proinflammatory cytokine that plays a pivotal role in the activation of various proinflammatory cytokines through signaling pathways. These include antimicrobial peptides that possess antifungal activity and neutrophil chemotactic factors (35). Th17 cells are a significant source of IL-17 during Candida infections. Genetic defects affecting the IL-17 pathway may impair the immune response against Candida, potentially leading to the progression of Chronic Mucocutaneous Candidiasis (CMC) disease (36).

## The relationship between CRAD9 and infection

### CARD9 and fungal infections

The deficiency of CARD9 renders individuals vulnerable to numerous fungal infections, including *Cryptococcus neoformans*, *Cryptococcus verrucosus*, *Aspergillus*, *Pneumocystis*, *Mucor irregularis*, *Rhizopus arrhizus*, among others (37–41). Susceptibility to fungal infections in patients lacking CARD9 is primarily attributed to impaired production of pro-inflammatory cytokines and chemokines, compromised neutrophil recruitment, and dysregulated activation of pathways such as NF-κB and MAPK. Additionally, deficiencies in Th-related responses, particularly in Th17 and Th22 cells, contribute to susceptibility. Furthermore, emerging evidence suggests that CARD9 plays regulatory roles in B cell-mediated humoral immunity, further emphasizing its significance in antifungal defense mechanisms (42).

### CARD9 and bacterial infections

The antimicrobial signaling cascade mediated by CARD9 is initiated by TLRs as part of the PRRs. Among TLR family members, TLR2 and TLR4 play pivotal roles in recognizing the components of gram-positive and gram-negative bacteria, respectively. A study indicates (42) that CARD9-mediated signaling is crucial for anti-pneumococcal immunity by regulating neutrophil function and cytokine production. Both neutrophil phagocytosis and accumulation depend on CARD9. Although CARD9 typically serves as a key downstream connector molecule for PRR-triggered signaling, it is important to note that PRRs can also initiate CARD9-independent antimicrobial signaling pathways. These pathways often intersect with the CARD9-dependent mechanisms to collectively exert bactericidal effects (43).

### CARD9 and Mycobacterium infections

The Dectin-1-Syk-CARD9 signaling pathway plays an important role in tuberculosis immunity (44, 45). Clecsf8 (MCL) is a FcRγ-coupled receptor (46). Cellular responses mediated by Clecsf8 depend on the Syk/CARD9 complex and encompass a spectrum of functions including phagocytosis, proinflammatory cytokine production, dendritic cell maturation, and T-cell initiation and bursts (47). Studies have demonstrated that Clecsf8-deficient mice are more susceptible to *Mycobacterium tuberculosis* infections. This susceptibility is characterized by increased load, hyperenhanced neutrophil infiltration, enhanced pathological injury, and early death. These findings underscore the critical role of the Clecsf8-Syk-CARD9 pathway in tuberculosis immunity and related immune responses (48). Similar Syk pathways are also present in *Mycobacterium bovis*, which promote the initiation of immune responses (49). Although *M. intracellulare* likely exhibits similarities, targeted research on its specific immune-mediated processes is lacking.

### CARD9 and viral infections

As a pivotal downstream molecule in the signal transduction of PRRs, CARD9 is increasingly being recognized for its significant role in viral infections. CARD9 integrates with the DNA sensor Rad50 and double-stranded DNA (dsDNA), leading to assembly of the dsDNA-Rad50-CARD9 complex. This complex ultimately triggers the activation of NF-κB and generation of a pro-IL-1β response to viral infections (50). Similar mechanisms have been observed for recognition of RNA viruses via the RIG-I pathway. RIG-I, along with the mitochondrial antiviral signaling proteins CARD9 and Bcl-10, activates NF-κB in response to RNA virus detection. These intricate signaling pathways highlight the multifaceted role of CARD9 in coordinating immune responses against both DNA and RNA viruses (51).

## Discussion

Overall, only 0.5% of invasive fungal infections involve CNS (52), with *Candida* meningitis being more common in newborns and patients using intracranial instruments (53, 54). High-risk factors primarily include abdominal surgery, intestinal perforation, recent broad-spectrum antibiotic treatment, intravenous drug use, extreme age, indwelling urinary catheters, and immunosuppression (AIDS, malignancy, antitumor treatment, and steroid use) (54). CARD9-deficient patients can eliminate *Candida* through neutrophil IgG opsonization, thereby preventing invasive *Candida* infections. However, when *Candida* appears in CNS compartments with low IgG levels, this defect may lead to chronic persistent *Candida* infection (55). One article reviewed 27 patients with invasive mycoses caused by various CARD9 mutations, primarily young individuals with a mean age of 22.1 years old. Of these, 58% were from Asia. *Candida* species accounted for 21 (78%) of the 27 cases responsible for cerebral infection, followed by *Trichophyton* species (11%), and *Exophiala dermatitidis* (3%). CNS infections manifested as cerebral abscesses (37%), meningoencephalitis (30%), meningitis (19%), and encephalitis (7%) (56). In clinical practice, it is widely believed that CARD9 deficiency is primarily associated with fungal infections, affecting various parts of the body, such as the skin, lungs, central nervous system, urinary tract, and others (17, 57–62). However, as mentioned earlier, it contributes to immune impairment against multiple infections. For instance, clinical reports have documented increased susceptibility to parasitic infections resulting from it (56). However, there is a notable scarcity of clinical reports on mycobacterial infections, particularly NTM infections. This study is the first clinical report highlighting the significant correlation between CARD9 deficiency and NTM infection globally, which serves as an impetus for completing this work.

This article presents a rare case of *C. albicans* combined with NTM CNS infection attributed to CARD9 deficiency in a middle-aged patient. We isolated and identified the specific type of NTM as *Mycobacterium intracellulare*. Through NGS, we identified the pathogen, and whole-exon sequencing helped elucidate the patient’s genetic defects. These findings suggest that clinicians should thoroughly consider immune-compromising factors when managing fungal infections in patients without HIV/AIDS, especially when IgE levels are elevated, suggesting the possibility of immunodeficiency. Furthermore, for immunocompromised individuals, emphasis should be placed on the heightened risk of multiple infections owing to their susceptibility to various pathogens. Given the varying detection rates of pathogens, particularly *Mycobacterium* with a detection rate of only 30–40% (63), employing multiple CSF cultures and utilizing novel technologies, such as NGS, are deemed necessary. These measures can enhance the accuracy of diagnosis and facilitate prompt and effective management of co-infections, thereby improving patient outcomes.

The treatment of invasive candidiasis necessitates the optimization of dosing regimens and an adequate course of therapy, considering factors such as the site of infection, species involved, patient risk factors, and pharmacokinetics. Currently, systemic antifungal therapy for invasive candidiasis comprises of 4 major classes: polyenes (including amphotericin B and its lipid-containing combinations), triazoles (such as fluconazole and voriconazole), echinocandins (including caspofungin and micafungin), and flucytosine. In cases of CNS candidiasis, treatment should be continued until symptoms, signs, cerebrospinal fluid abnormalities, and head imaging abnormalities are resolve (64). The utilization of granulocyte-macrophage colony-stimulating factor (GM-CSF) and granulocyte colony-stimulating factor (G-CSF) as immunoadjuvant therapies in conjunction with stem cell transplantation has shown promising results in patients with CARD9 gene defects (65). Currently, there is no consensus on antifungal treatment strategies for patients with CARD9 deficiency. Some stress the importance of lifelong antifungal prophylaxis (66), while others advocate secondary prevention using oral azole drugs following the initial episode of invasive fungal disease (IFD) (15). In cases where there is no recurrence during treatment, long-term (at least 9–12 months) therapy should be considered (15). In this article, the patient’s antifungal treatment was extended beyond 9 months, with medication cessation occurring following the observation of normal CSF and head MRI results. Subsequent follow-up examinations confirmed the efficacy of this treatment regimen. However, there are currently insufficient high-quality evidence-based medical data to advocate the necessity of long-term or lifelong administration of antifungal drugs to prevent recurrence in patients with CARD9 gene defects. Considering that over 85% of CARD9 deficiency patients experience recurrence of fungal disease upon treatment (15), clinical practitioners should prioritize long-term follow-up and not overlook this aspect. A previous report (67) described that identical twins with CARD9 deficiency both suffered from severe CNS *C. albicans* infections. However, in this study, the patient and his homozygous brother are not twins. It was learned from the patient that his brother does not appear to have severe CNS infections, which may be related to genetic heterogeneity. We attempted to perform comprehensive and systematic examinations on his brother, but he refused.

The optimal drug dosage, combination, and duration for treating most NTM diseases have yet to be determined, and combination therapy is recommended, although its efficacy in clinical practice can vary. Treatment protocols recommended by the American Thoracic Society and the British Thoracic Society (68, 69) for disseminated MAC disease primarily involve ethambutol and clarithromycin (or azithromycin), with or without rifabutin, continuing until 1 year after achieving culture negativity. Although drug sensitivity testing is valuable, personalized treatment selection remains crucial. In this case, despite rifampin’s sensitivity in drug sensitivity testing, we opted not to use it because of concerns that it may not reach the minimum inhibitory concentration in CNS (70). Instead, we decided to include moxifloxacin in addition to ethambutol and clarithromycin as intensive treatment, which ultimately led to a successful clinical cure.

Overall, the diagnosis and treatment of this patient were timely, accurate, and effective. However, when reviewing the entire course of the disease, we still identified some limitations: during the second hospitalization, when NTM positivity was reported, we did not provide appropriate treatment in a timely manner. Although considering the reason of unclear drug susceptibility results, it may led to an extension of the patient’s disease course; Secondly, the follow-up time of this patient after stopping the treatment was still insufficient. We need further long-term follow-up to determine the effectiveness of the treatment.

### Limitations

1. We did not conduct an in-depth investigation into the protein alterations caused by this gene mutation.
2. Due to time and financial constraints, the patient failed to complete the detection of cytokines.

## Conclusion

In summary, c.175C>T (p. Arg59Trp) is a novel CARD9 gene mutation and is probably damaging. *C. albicans* meningitis is uncommon in clinical practice, and co-occurrence of NTM infections is even rarer. For clinical diagnosis and treatment, pathogenetic tests, such as whole blood and CSF analyses, should be performed promptly. The early implementation of NGS can facilitate early detection, diagnosis, and treatment, thereby reducing mortality. During treatment, adherence to standardized antifungal treatment guidelines is critical. In addition, treatment should be personalized for patients with long-term or persistent disease, particularly for those without HIV/AIDS. It is crucial to explore the etiology of this condition, and whole-exome sequencing should be considered to identify potential gene deletions.

## Data Availability

The original contributions presented in the study are included in the article/supplementary material. Further inquiries can be directed to the corresponding author.
